# Experimental data of cathodes manufactured in a convective dryer at the pilot-plant scale, and charge and discharge capacities of half-coin lithium-ion cells

**DOI:** 10.1016/j.dib.2021.107720

**Published:** 2021-12-16

**Authors:** Luis A. Román-Ramírez, Geanina Apachitei, Mona Faraji-Niri, Michael Lain, Dhammika Widanage, James Marco

**Affiliations:** a Warwick Manufacturing Group, University of Warwick, Coventry CV4 7AL, UK; b London South Bank University, 103 Borough Road, London, SE1 0AA, UK; c The Faraday Institution, Quad One, Harwell Science and Innovation Campus, Didcot, UK

**Keywords:** Lithium-ion battery, Manufacturing, Cathode, Coating, Drying, Electrochemical testing, Capacity, Experimental data

## Abstract

Megtec Systems pilot-plant scale continuous convective coater. The data was generated as part of an experimental design involving the following coating-drying process variables and ranges: comma bar gap, 80–140 µm; web speed, 0.5–1.5 m/min; coating ratio, 110–150%; drying temperature, 85–110 °C and drying air speed, 5–15 m/s. The manufacturing data include pre-calendered coating thickness, mass loading dry and wet, pre-calendered porosity, spatial autocorrelation and join counting (SAJC) *Z*-score for carbon and for fluorine, cell thickness, coating weight and porosity of 15 different electrode coatings and 45 half-coin cells. The electrochemical data was obtained at 25 °C in a Maccor 4000 series battery cycler and consists of charge and discharge capacities at C/20, C/5, C/2, 1C, 2C, 5C and 10C C-rates. Discharge gravimetric and volumetric capacities, rate performance (at 5C:0.2C) and first cycle loss data is also reported. Details of the experimental design and a comprehensive analysis of the data can be found in the co-submitted manuscript (Román-Ramírez et al., 2021). Additional collected data not used in Román-Ramírez et al. (2021) is reported in the present manuscript and include visual observations of coating defects, rheological properties of the electrode slurries (solid content, viscosity, coating shear rate and viscosity at coating shear rate), room temperature and room humidity during the coatings and first cycle loss of the coin cells. Raw and analyzed data is made available. The reported data can be used to extend the analysis reported in Román-Ramírez et al. (2021), and for the comparison of relevant data obtained at different manufacturing scales.

**Specifications Table**

**Table:** 

Subject	Electrical and Electronic Engineering
Specific subject area	Electrode manufacturing and characterization of lithium-ion batteries
Type of data	Table Image Excel files (.xlxs) Maccor files (.csv) Biologic files (.mpt)
How data were acquired	Electrode and cell manufacturing data • Coating and drying of the electrodes were performed in a Megtech Systems pilot-plant convective coater. • The mass loadings wet and dry were recorded by MeSys GmbH system. Data processing was done in MATLAB (2020b). • The porosities of the pre-calendered and calendered (strips) electrodes are calculated values according to the equations reported in Section 2.1). • Hegman gauge (fineness of grind) was used to check the presence of large particles or clusters. • The coating thicknesses of the pre-calendered and calendered (strips) were measured by a digital thickness gauge (Mitutoyo). • Carbon and fluorine distribution were determined by spatial autocorrelation and join counting (SAJC) *Z*-score as detailed in Section 2.1. • Scanning electron microscope (SEM) images were obtained from a Hitachi TM3030 microscope fitted with a 30 mm^2^ energy dispersive X-ray spectroscopy (EDS) detector (Oxford Instruments). Half-coin cell physical measurements • The thicknesses were measured by a digital thickness gauge (Mitutoyo). • The porosities are calculated values according to the equations in Section 2.1. • Rheological properties ○Solid content was determined by a moisture analyzer (Ohaus, MB120). ○Viscosity vs shear rate data was determined in a rotational rheometer (Anton Paar). • Room temperature and humidity are readings from a Thermo-Hygro Sensor (Oregon Scientific). Electrochemical testing and determinations • Charge and discharge capacities of 39 coin cells were obtained in a Maccor 4000 series battery cycler. Six coin cells were measured in a Biologic BCS-805 cycler.
	Gravimetric capacities, volumetric capacities, rate performance and first cycle loss were calculated according to the equations in Section 2.2. Rate capacity coefficients are calculated values as described in Section 2.2.
Data format	Raw Analyzed
Parameters for data collection	The manufacturing data was generated as part of a Design of Experiments (DoE) study consisting of 12 experimental runs as detailed in Section 2.1. The electrochemical data was obtained in a temperature-controlled chamber at 25 °C using the Maccor battery cycler with a maximum channel charge current of 5 A. The testing protocol involved upper and lower cut-off voltages of 4.2 V and 2.5 V, respectively. Formation cycle was performed at C/20 rate, followed by five conditioning cycles at C/5. Discharge C-rate capacities were measured at C/20, C/5, C/2, 1C, 2C, 5C and 10C with all charging cycles done at C/5. Similar conditions were used for the Biologic battery cycler.
Description of data collection	Rheological measurements were recorded manually. Coating conditions (comma bar gap, coating ratio, web speed, drying air temperature and drying air speed) were set and recorded by the Megtech Systems. Mass loadings dry and wet were recorded by the MeSys GmbH system. The porosities were computed according to the equations in Section 2.1. Coating thicknesses were recorded manually. SEM and EDS images were collected from the electronic microscope software. Electrochemical testing data was collected from .cvs output files from the Maccor software, or .mpt files from Biologic software.
Data source location	Institution: Warwick Manufacturing Group (WMG). University of Warwick City: Coventry Country: United Kingdom GPS coordinates for collected samples/data: 52.38363378953185, −1.5615186436655097
Data accessibility	Dataset available as supplementary files.
Related research article	Román-Ramírez LA, Apachitei G, Faraji Niri M, Lain M, Widanage D, Marco J. Understanding the effect of coating-drying operating variables on electrode physical and electrochemical properties of lithium-ion batteries. J Power Sources 2021;516. doi:10.1016/j.jpowsour.2021.230689.

**Value of the Data**

• The dataset contains operating conditions of the coating-drying manufacturing process of NMC6222 cathodes at a pilot-plant scale, physical properties of the resulting electrodes and cells, and electrochemical properties of half-coin cells. Rheological data and room conditions during the process is also reported which can be valuable in studies looking at the effect of such variables on electrode structure or cell performance. The raw electrochemical data can be valuable in the evaluation of cells manufactured with a different formulation or operating conditions.
• Academic and industrial researchers interested in the manufacturing, testing or model development of lithium-ion cells can benefit from the data.
• The dataset can be compared with manufacturing data acquired at a laboratory or industrial scale to gain further insights on manufacturing scales and cell performance. The data can also be used to compare new manufacturing technologies of lithium-ion batteries. The electrochemical data can be used for model development, testing and/or validation of electrochemical models.

## 1 Data Description

### 1.1 Electrode manufacturing data

The Excel file *Operating variables and responses.xlsx* available within the supplementary files, contains the set of experimental conditions (coater machine settings) used to collect the data, the measured responses and the standard deviations of the measurements. More specifically, the Excel sheet *Factors and levels* within *Operating variables and responses.xlsx* contains a summary of the operating variables studied and their settings. The *Design matrix and Responses* sheet within *Operating variables and responses.xlsx* presents the design matrix (set of experiments) according to the experimental design outlined in Section 2.1, and the values of the responses for each of the 12 experimental runs in the design matrix. The *Design matrix and Responses* sheet also presents the operating conditions and values of the responses for 3 validation runs. The experiments are labeled as NEX_CATX_Y, where *X* refers to a slurry batch number and *Y* to the experimental run number.

The set of reported responses include:

• Visual coating defects
• Spatial Autocorrelation (Carbon) Join Count *Z* Score
• Spatial Autocorrelation (Fluorine) Join Count *Z* Score
• Pre-calendered coating thickness
• Pre-calendered porosity
• Mass loading wet
• Mass loading dry
• Calendered thickness
• Calendered porosity
• Cell thickness
• Cell coating weight
• Cell porosity

The *Design matrix and Responses* sheet also contains the rheological properties of the slurries used for the coatings, and the room temperature and room humidity conditions. The slurry rheological properties reported are:

• Slurry solid content
• Slurry viscosity at 10 s^−1^
• Coating shear rate
• Viscosity at coating shear rate

The *Standard deviations* sheet within *Operating variables and responses.xlsx* contains the standard deviations for all the measurements carried out.

Coating conditions and wet and dry mass loading recordings from the Megtec and MeSys Sytems can be found in the *Megtech and Mesys recordings* folder within the supplementary files. The recordings are in the form of raw .xlsx files.

Pictures of the Hegman gauge (fineness of grind) determinations can be found in the *Hegman gauge* folder, available within the supplementary files.

Scanning electron microscope (SEM) and energy dispersive X-ray spectroscopy (EDS) images of the pre-calendered electrodes can be found in the *SEM and EDS images* folder, available within the supplementary files.

Spatial autocorrelation and join counting (SAJC) *Z*-score were determined From the EDS maps for carbon and fluorine following the procedure described in Section 2.1. The values of the SAJC *Z*-score for carbon and fluorine are shown in the *Design matrix and Responses* sheet.

### 1.2 Electrochemical data

The electrochemical data can be found in the *Design matrix and Responses*, and include the following determinations:

• Gravimetric capacities at C/20, C/5, C/2, 1C, 2C, 5C and 10C rate
• Volumetric capacities at C/20, C/5, C/2, 1C, 2C, 5C and 10C rate
• Rate performance at 5C:C/5
• First cycle loss
• Rate capacity coefficient, κ

The reported electrochemical data was calculated from discharge capacity measurements and half-coin cell physical properties according to the equations and procedures detailed in Section 2.2. Charge and discharge capacities, cell physical characteristics and testing conditions data of the individual coin cells for each of the experimental runs can be found in the *Half-coin cells data.xlsx* file in the supplementary files. The Excel sheets in the *Half-coin cells data* file are labeled according to the NEX_CATX_Y notation described in Section 1.1, and contain the data for three coin cells for the corresponding experimental run. The following data is available for each individual cell:

• Assembly date
• Active material
• Dry composition
• Capacity of active material (powder form)
• Coated foil mass
• Bare foil mass
• Foil thickness
• Total thickness
• Area
• Theoretical density at 0% porosity
• Voltage after assembly
• Voltage open circuit on testing equipment
• Testing equipment
• Coating mass
• Expected capacity
• Coating thickness
• Coating weight
• Density
• Porosity
• Capacity charge at: C/20, C/5 (11 cycles)
• Capacity discharge at: C/20, C/5 (6 cycles), C/2, C, 2C, 5C and 10C
• Calculated gravimetric and volumetric capacities at the different C-rates
• Rate performance at 5C:0.2C
• First cycle loss

The average and standard deviations from the measurements of three coin cells are also shown in the *Half-coin cells data.xlsx* file.

Raw capacity data in the form of Maccor.csv files for the individual cells can be found in the *Maccor* folder within the supplementary files. The Biologic.mpt files can be found in the *Biologic* folder within the supplementary files.

## 2 Experimental Design, Materials and Methods

### 2.1 Electrode and cell manufacturing data

The set of experiments were determined based on a Placket-Burman (PB) design [2]. The factors (operating variables) and levels (settings) for the PB design, as well as the resulting design matrix (set of experiments) can be found in Table 1 of the related research article [1].

Visual coating defects of the electrodes were determined based on simple visual inspection for fisheyes, agglomerates, streaks, cracks, wet coating, etc.

The working principle of the MeSys systems used for recording the wet and dry mass loading is: the material under investigation is locally oscillated; the variations in the oscillation reflection and transmission are detected and carry information about the mass loading (grammage) of the material. The two scanners (for wet coating- before the electrode enters the oven and for dry coating- when it exits the oven) are continuously moving across the foil, creating a zig-zag acquisition pattern when the web is moving through the coater. The system is similar to the ones used in industry scale manufacturing facilities.

Mass loadings wet and dry were obtained from recordings of the MeSys Systems. The mass loading data are two dimensional with time and position of the recording sensor of the MeSys system. The mass loading recordings include the weight of bare foil and also non-uniform at the edge of the coatings. In order to polish this data, the following steps were taken.

1. The bare foil weight was subtracted from the whole data, for Cathode the bare foil weight was 90 (g/cm^2^).
2. The data were trimmed to remove the non-uniform edges, this was performed by identifying the edges after visualizing the 2D data in MATLAB.
3. The trimmed data were then searched to locate missing values which were unavoidable due to the equipment error, the missing values were consequently replaced by the mean value of the recordings at the same location but in other time instants.
4. In the next step the data were searched to detect the outliers. Outliers were defined as data points at least three times of the scaled median absolute deviations away from the median of that recording of that experiment. The outlier points were then replaced with the mean value of the nearest neighbor (non-outlier) data points. This was to keep the dataset continuous as the data were related to a 2D plane.
5. Finally, feature extraction was performed where the mean, standard deviation and median of data were calculated.

The MATLAB script file can be made available upon request from the corresponding authors. Due to recording issues, the mass loading wets for the experiments 5 to 12 are not available. These values were substituted with display readings from the MeSys Systems during the experiment.

Calendering was done in a medium laboratory calender, with 203 mm width and 203 mm diameter rolls. The operating parameters of the calendaring process were kept constant for all coatings: 85 °C and 0.5 m/min roll speed. The parameter that was changed is the gap between the calender rolls which translates into the applied pressure during calendering. The electrode was coated with a 9 cm width and cut into 15 cm wide sheets for further processing. The initial thickness of every electrode sheet was measured in up to 10 points, producing the mean and standard deviation. The dry coating weight reading from the MeSys systems and the measured thickness was used in the calculation of porosity (P) according to Eq. (1). The electrodes were then passed through the calender rolls and the resulting thickness measured. The process was repeated until one of the following occurred: (a) the target value of the porosity (30% ± 1%) was reached; (b) keeping the same gap would not produce a thickness decrease, but using a lower gap would jam the calendar. This experimental limit resulted in obtaining porosities up to 32% for the calendered sheets.

(1) $$P=1-\frac{\frac{dry\;mass\;loading}{coating\;thickness}}{\rho_{bulk}}$$

In Eq. (1), the dry mass loading is the value read from the MeSys system, the coating thickness is the total thickness after extracting the current collector thickness. The density of the coating $\rho_{bulk},\;$is the density of the components at 0% porosity, 4.53 g/cm^3^.

The reported thicknesses are the average values of 10 measurement at different locations on a 9 cm × 15 cm electrode (pre-calendared or calendered) or electrode disc used in the coin cells.

The thicknesses of the pre-calendered and calendered electrodes (strips/sheets), and the coin cells (electrode discs) were measured by a digital thickness gauge (Mitutoyo) with a precision of 1 µm.

The porosities of the calendered electrodes and the cells were computed using Eq. (2), where $\rho_{coating}$ is the effective density of the coating and $\rho_{bulk}$ is the approximation of the coating density at 0% porosity. The coating mass loading, m, and thickness, $t_{coating}$, can be used to define the coating density.

(2) $$P=1-\frac{\rho_{coating}}{\rho_{bulk}}=1-\frac{m}{t_{coating\;}\cdot \rho_{bulk}}$$

SEM and EDS images, and SAJC *Z*-Score for carbon and fluorine were determined using the following procedure: small disks of the coatings were microtomed, to give a clean edge for analysis. After cutting, the disks were mounted on metal strips with conductive carbon tape, and then fixed in SEM stubs with a vertical slot. SEM images and EDS maps were recorded using a desktop scanning electron microscope (SEM, Hitachi TM3030), fitted with a 30 mm^2^ energy dispersive X-ray spectroscopy (EDS) detector (Oxford Instruments). The region used for the EDS map was chosen to be as representative of the coating as possible. After the analysis, the EDS maps were converted to individual *.jpg files, which were cropped to the region of the coating. The images were converted from color to black and white, and the contrast was maximized. The *.jpg files were then converted to *.txt files using ImageJ software [3]. Finally, these files were imported into an Excel spreadsheet, to perform the join counting calculations. Each pixel had a value between 0 (black) and 255 (white). The threshold to convert these numbers into binary values was in the range 100–130. The threshold values were adjusted to give a surface coverage percentage for which expected mean and standard deviation values had been calculated. The equation used to calculate the Z_1-1_ join counting score is given in [1].

The slurry solid content was measured using a moisture analyzer (Ohaus, MB120). The principle is to spread a thin layer of slurry and heat it until the solvent evaporates completely. The remaining mass should illustrate the solids in the slurry. The viscosity at the coating shear rate was determined from slurry vs shear rate data obtained in a rotational rheometer (Anton Paar) in a concentric cylinder measuring system set CC27 (27 mm diameter cylinder with smooth finish). The slurry was allowed to stabilize at 25 °C before starting the procedure.

Room temperature and room humidity were taken from visual readings of a thermo-hygro sensor (Oregon Scientific). The values would reflect the environmental conditions where the mixing and coating have been performed.

### 2.2 Electrochemical data

The electrochemical testing involved obtaining discharge C-rate capacities on three coin cells produced from the same coating (experiment) to capture cell-to-cell variation. The reported values of the test are the average and the standard deviations computed from the three coin cell measurements. The electrochemical tests were performed in a temperature-controlled chamber at 25 °C using a Maccor 4000 series battery cycler with maximum channel charge current of 5 A. The testing protocol was created with upper and lower cut-off voltages of 4.2 V and 2.5 V, respectively. Formation cycle was performed at C/20 rate, followed by five conditioning cycles at C/5. Discharge C-rate capacities were measured at C/20, C/5, C/2, 1C, 2C, 5C and 10C with all charging cycles done at C/5. The data was exported into a .csv file. The coin cells for the validation experiments 2 and 3 (see *Design matrix and Responses.xlsx* file) were tested in a Biologic BCS-805 following the same testing protocol used for Maccor. Gravimetric and volumetric capacities were then calculated from Eqs. (3) and (4), respectively. Rate performance and First cycle loss were defined according to Eqs. (5) and (6), respectively.

The rate capacity coefficient, κ, was determined as the gradient of the plot of Ln(discharge capacity) vs C-rate.

(3) $$Gravimetric\;capacity=\frac{capacity}{\left(electrode\;mass-foil\;mass\right)\left(active\;material\;wt\%\right)}$$

(4) $$Volumetric\;capacity=\frac{capacity}{cell\;area\;\left(total\;thickness-foil\;thickness\right)}$$

(5) $$Rate\;performance\;at\;5C:0.2C=\left(\frac{discharge\;capacity\;at\;5C}{discharge\;capacity\;at\;C/5}\right)100$$

(6) $$First\;cycle\;loss=\left(\frac{charge\;capacity\;at\;C/20-discharge\;capacity\;at\;C/20}{charge\;capacity\;at\;C/20}\right)100$$

## Ethics Statement

Not applicable.

## CRediT Author Statement

GA collected the manufacturing data and the electrochemical data. GA, ML and LAR processed the electrochemical data for dissemination and analysis. GA and LAR planned the experimental campaign. MFN processed the MeSys data. ML collected and processed the SEM, EDS and SAJC data. LAR wrote the initial draft. JM and DW reviewed and edited the article. JM directed the research.

## Declaration of Competing Interest

The authors declare that they have no known competing financial interests or personal relationships which have or could be perceived to have influenced the work reported in this article.

## Appendix Supplementary materials

Image, application 1
