# Supplementary figures and images for: Experimental data of cathodes manufactured in a convective dryer at the pilot-plant scale, and charge and discharge capacities of half-coin lithium-ion cells
Source: Data Brief. 2021 Dec 16;40:107720. doi: 10.1016/j.dib.2021.107720 (PMC8703052; doi:10.1016/j.dib.2021.107720)

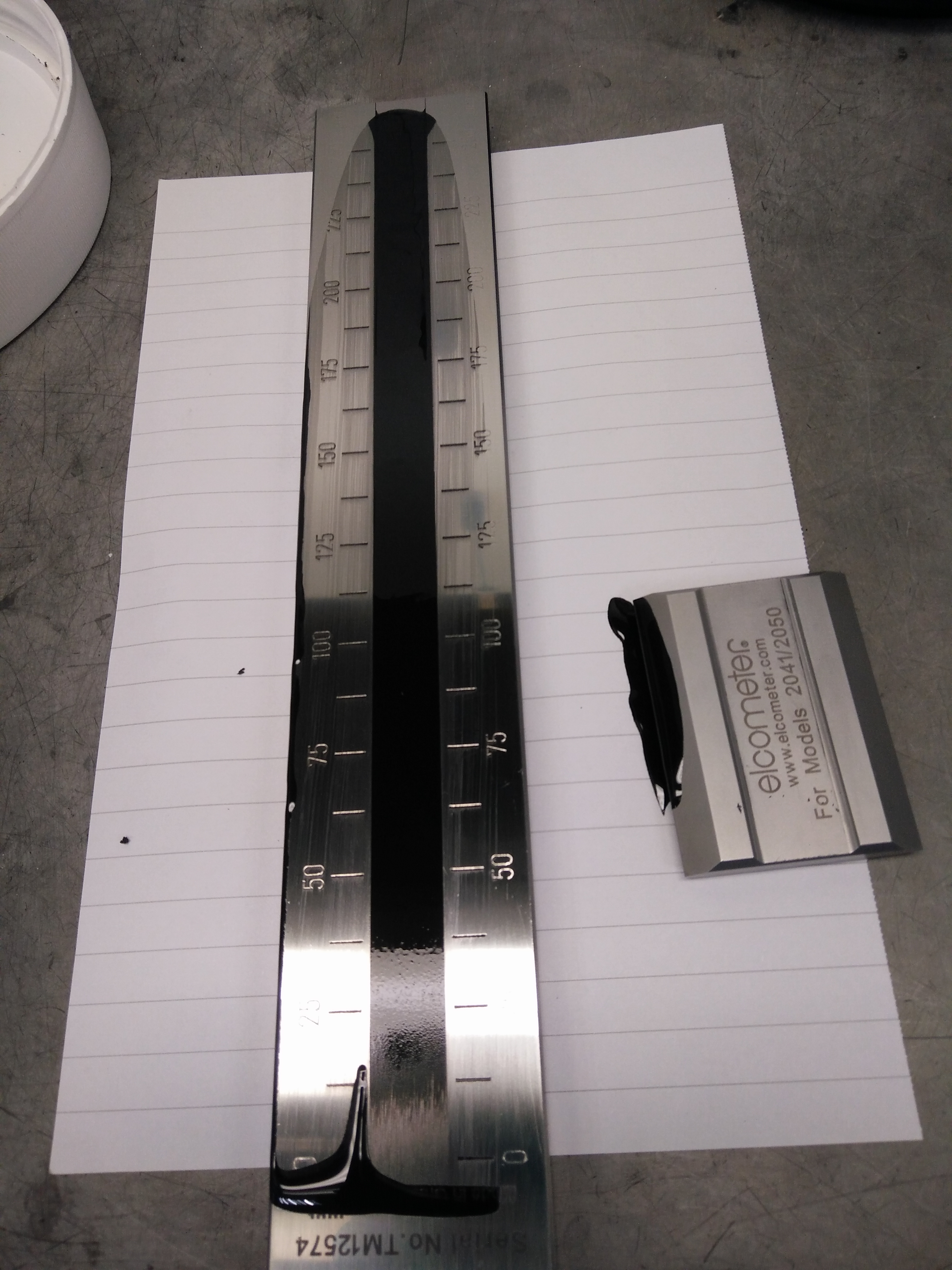

Supplement: Supplementary file 1 [file mmc1.zip › Hegman gauge/Hegman 1.jpg]

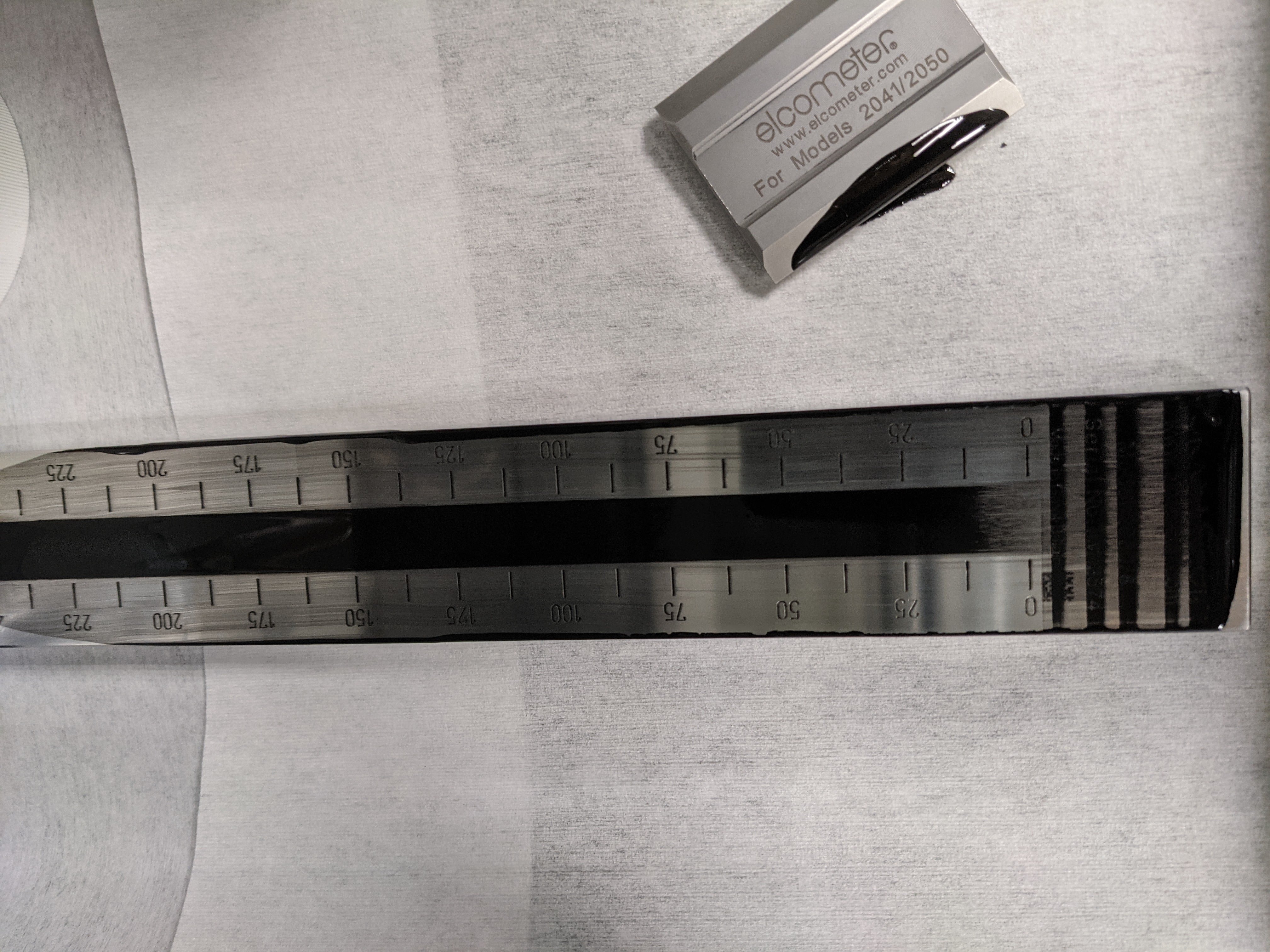

Supplement: Supplementary file 1 [file mmc1.zip › Hegman gauge/Hegman 2.jpg]

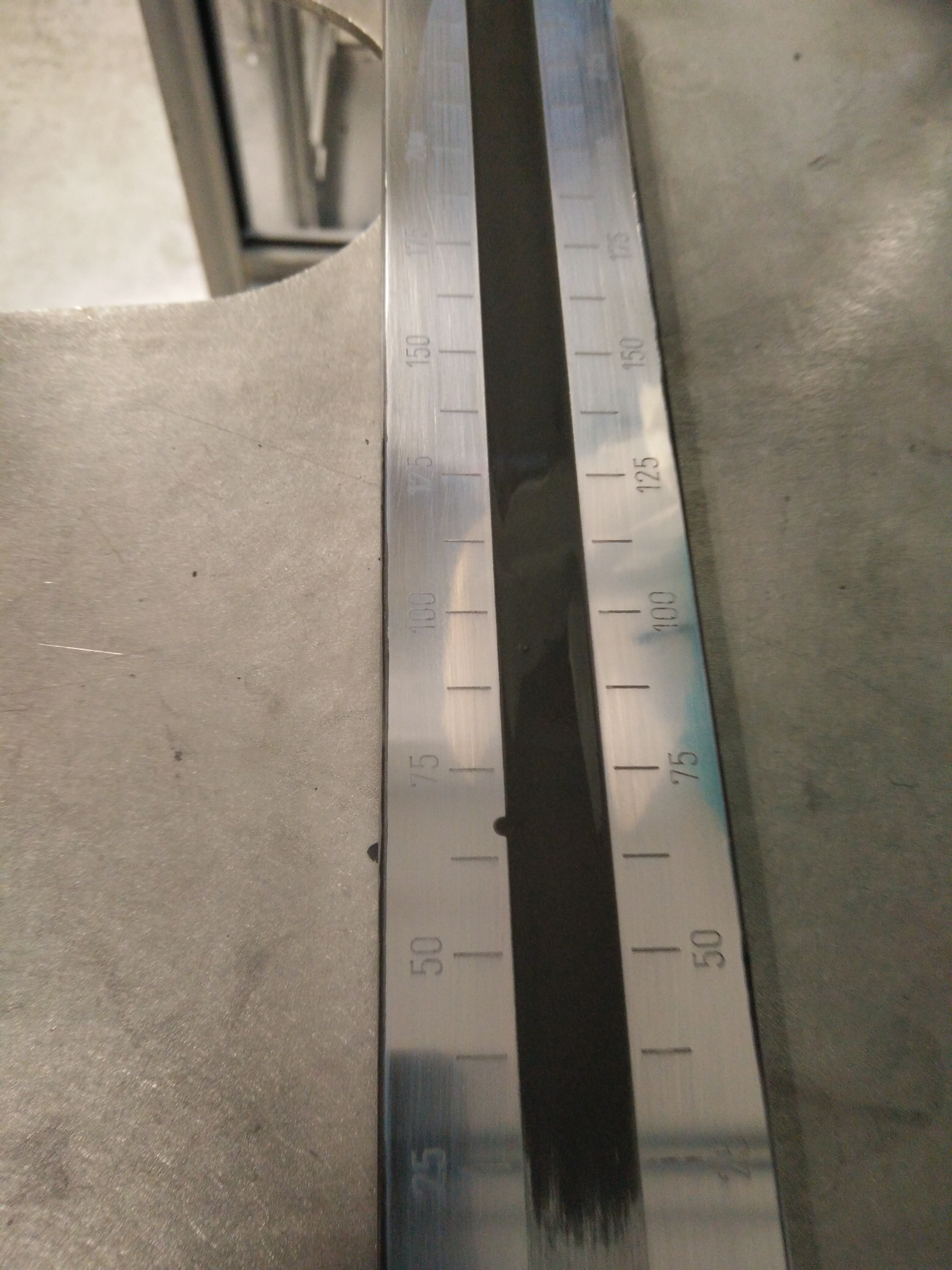

Supplement: Supplementary file 1 [file mmc1.zip › Hegman gauge/Hegman 3.jpg]

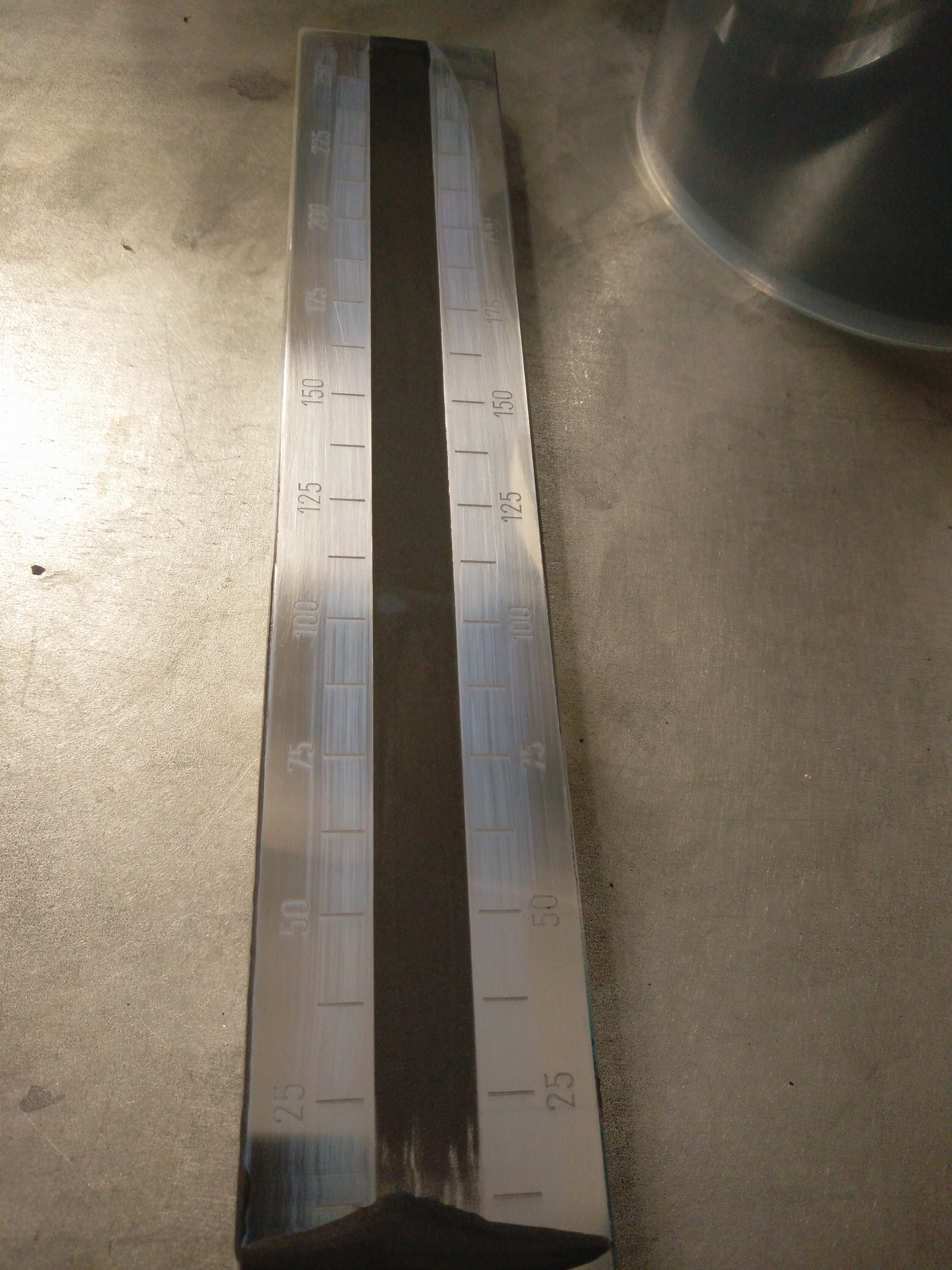

Supplement: Supplementary file 1 [file mmc1.zip › Hegman gauge/Hegman 4.jpg]

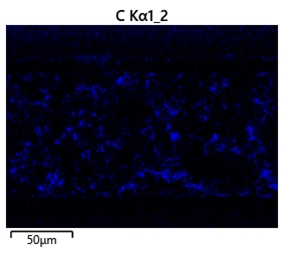

Supplement: Supplementary file 1 [file mmc1.zip › SEM and EDS images/CAT2-03 C.jpg]

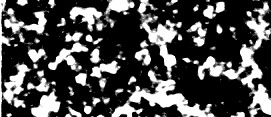

Supplement: Supplementary file 1 [file mmc1.zip › SEM and EDS images/CAT2-03 CY.jpg]

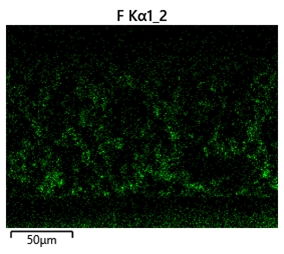

Supplement: Supplementary file 1 [file mmc1.zip › SEM and EDS images/CAT2-03 F.jpg]

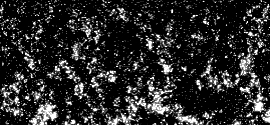

Supplement: Supplementary file 1 [file mmc1.zip › SEM and EDS images/CAT2-03 FY.jpg]

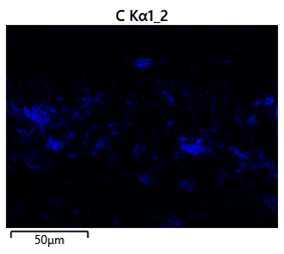

Supplement: Supplementary file 1 [file mmc1.zip › SEM and EDS images/CAT2-04 C.jpg]

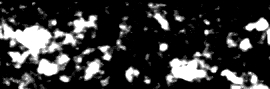

Supplement: Supplementary file 1 [file mmc1.zip › SEM and EDS images/CAT2-04 CY.jpg]

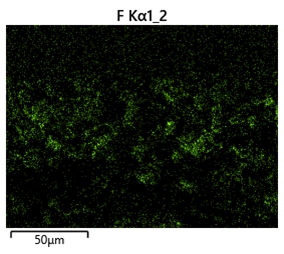

Supplement: Supplementary file 1 [file mmc1.zip › SEM and EDS images/CAT2-04 F.jpg]

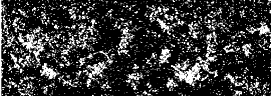

Supplement: Supplementary file 1 [file mmc1.zip › SEM and EDS images/CAT2-04 FY.jpg]

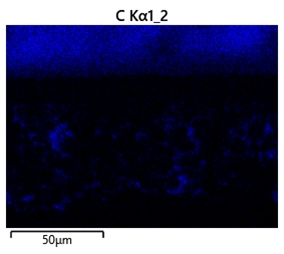

Supplement: Supplementary file 1 [file mmc1.zip › SEM and EDS images/CAT2-05 C.jpg]

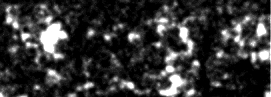

Supplement: Supplementary file 1 [file mmc1.zip › SEM and EDS images/CAT2-05 CY.jpg]

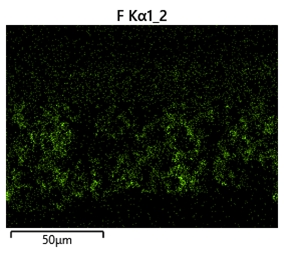

Supplement: Supplementary file 1 [file mmc1.zip › SEM and EDS images/CAT2-05 F.jpg]

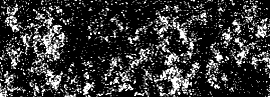

Supplement: Supplementary file 1 [file mmc1.zip › SEM and EDS images/CAT2-05 FY.jpg]

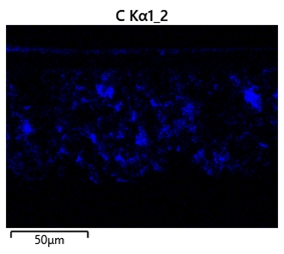

Supplement: Supplementary file 1 [file mmc1.zip › SEM and EDS images/CAT2-06 C.jpg]

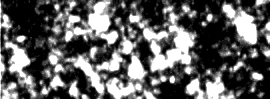

Supplement: Supplementary file 1 [file mmc1.zip › SEM and EDS images/CAT2-06 CY.jpg]

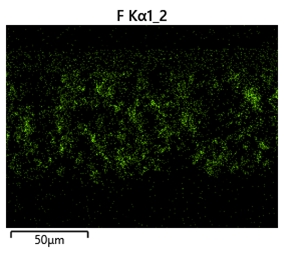

Supplement: Supplementary file 1 [file mmc1.zip › SEM and EDS images/CAT2-06 F.jpg]

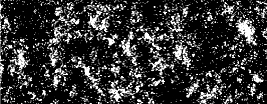

Supplement: Supplementary file 1 [file mmc1.zip › SEM and EDS images/CAT2-06 FY.jpg]

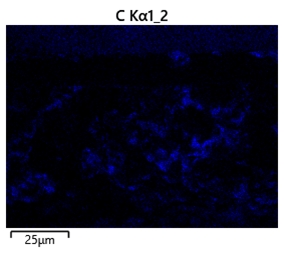

Supplement: Supplementary file 1 [file mmc1.zip › SEM and EDS images/CAT3-07 C.jpg]

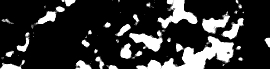

Supplement: Supplementary file 1 [file mmc1.zip › SEM and EDS images/CAT3-07 CY.jpg]

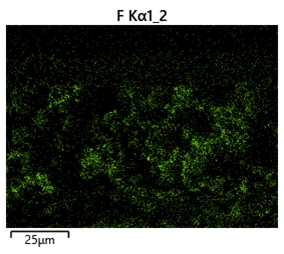

Supplement: Supplementary file 1 [file mmc1.zip › SEM and EDS images/CAT3-07 F.jpg]

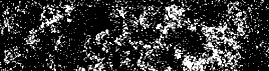

Supplement: Supplementary file 1 [file mmc1.zip › SEM and EDS images/CAT3-07 FY.jpg]

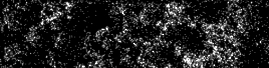

Supplement: Supplementary file 1 [file mmc1.zip › SEM and EDS images/CAT3-07 FYY.jpg]

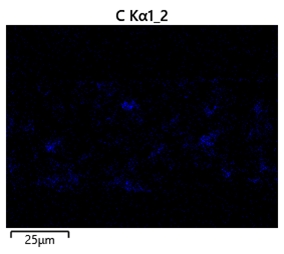

Supplement: Supplementary file 1 [file mmc1.zip › SEM and EDS images/CAT3-08 C.jpg]

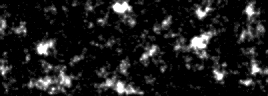

Supplement: Supplementary file 1 [file mmc1.zip › SEM and EDS images/CAT3-08 CY.jpg]

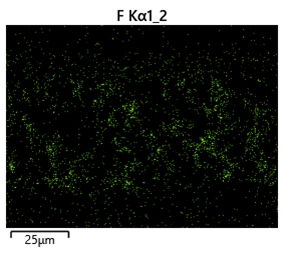

Supplement: Supplementary file 1 [file mmc1.zip › SEM and EDS images/CAT3-08 F.jpg]

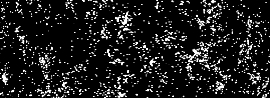

Supplement: Supplementary file 1 [file mmc1.zip › SEM and EDS images/CAT3-08 FY.jpg]

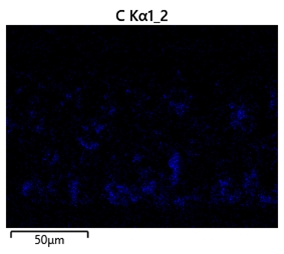

Supplement: Supplementary file 1 [file mmc1.zip › SEM and EDS images/CAT3-09 C.jpg]

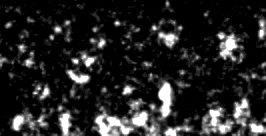

Supplement: Supplementary file 1 [file mmc1.zip › SEM and EDS images/CAT3-09 CY.jpg]

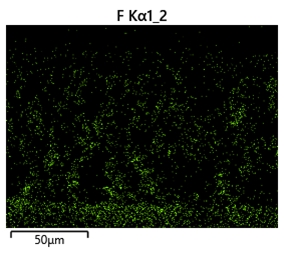

Supplement: Supplementary file 1 [file mmc1.zip › SEM and EDS images/CAT3-09 F.jpg]

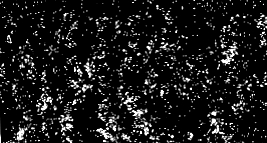

Supplement: Supplementary file 1 [file mmc1.zip › SEM and EDS images/CAT3-09 FY.jpg]

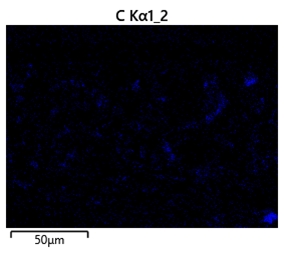

Supplement: Supplementary file 1 [file mmc1.zip › SEM and EDS images/CAT3-10 C.jpg]

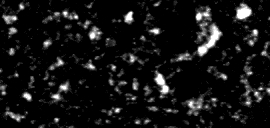

Supplement: Supplementary file 1 [file mmc1.zip › SEM and EDS images/CAT3-10 CY.jpg]

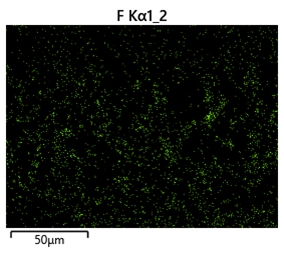

Supplement: Supplementary file 1 [file mmc1.zip › SEM and EDS images/CAT3-10 F.jpg]

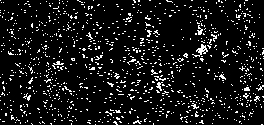

Supplement: Supplementary file 1 [file mmc1.zip › SEM and EDS images/CAT3-10 FY.jpg]

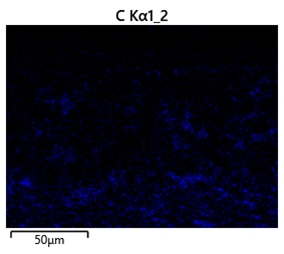

Supplement: Supplementary file 1 [file mmc1.zip › SEM and EDS images/CAT3-11 C.jpg]

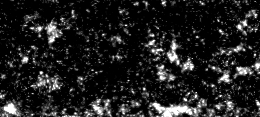

Supplement: Supplementary file 1 [file mmc1.zip › SEM and EDS images/CAT3-11 CYY.jpg]

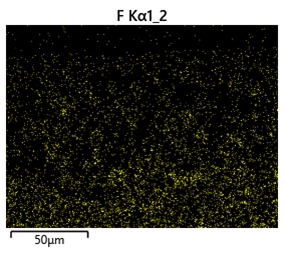

Supplement: Supplementary file 1 [file mmc1.zip › SEM and EDS images/CAT3-11 F.jpg]

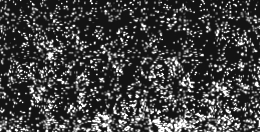

Supplement: Supplementary file 1 [file mmc1.zip › SEM and EDS images/CAT3-11 FYY.jpg]

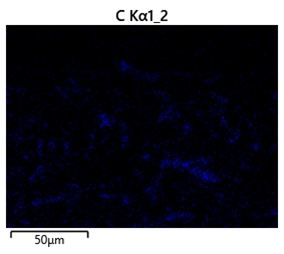

Supplement: Supplementary file 1 [file mmc1.zip › SEM and EDS images/CAT3-12 C.jpg]

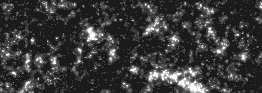

Supplement: Supplementary file 1 [file mmc1.zip › SEM and EDS images/CAT3-12 CYY.jpg]

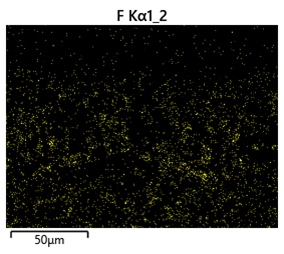

Supplement: Supplementary file 1 [file mmc1.zip › SEM and EDS images/CAT3-12 F.jpg]

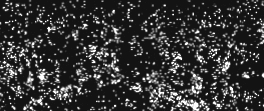

Supplement: Supplementary file 1 [file mmc1.zip › SEM and EDS images/CAT3-12 FY.jpg]

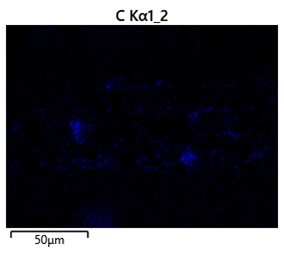

Supplement: Supplementary file 1 [file mmc1.zip › SEM and EDS images/CAT3-13 C.jpg]

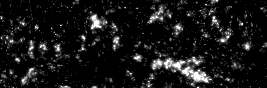

Supplement: Supplementary file 1 [file mmc1.zip › SEM and EDS images/CAT3-13 CYY.jpg]

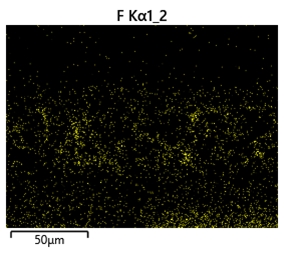

Supplement: Supplementary file 1 [file mmc1.zip › SEM and EDS images/CAT3-13 F.jpg]

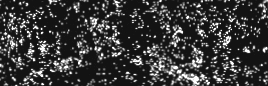

Supplement: Supplementary file 1 [file mmc1.zip › SEM and EDS images/CAT3-13 FYY.jpg]

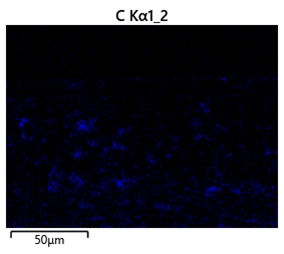

Supplement: Supplementary file 1 [file mmc1.zip › SEM and EDS images/CAT3-14 C.jpg]

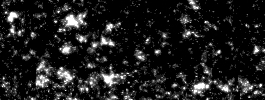

Supplement: Supplementary file 1 [file mmc1.zip › SEM and EDS images/CAT3-14 CYY.jpg]

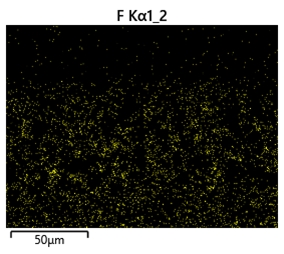

Supplement: Supplementary file 1 [file mmc1.zip › SEM and EDS images/CAT3-14 F.jpg]

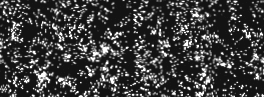

Supplement: Supplementary file 1 [file mmc1.zip › SEM and EDS images/CAT3-14 FYY.jpg]

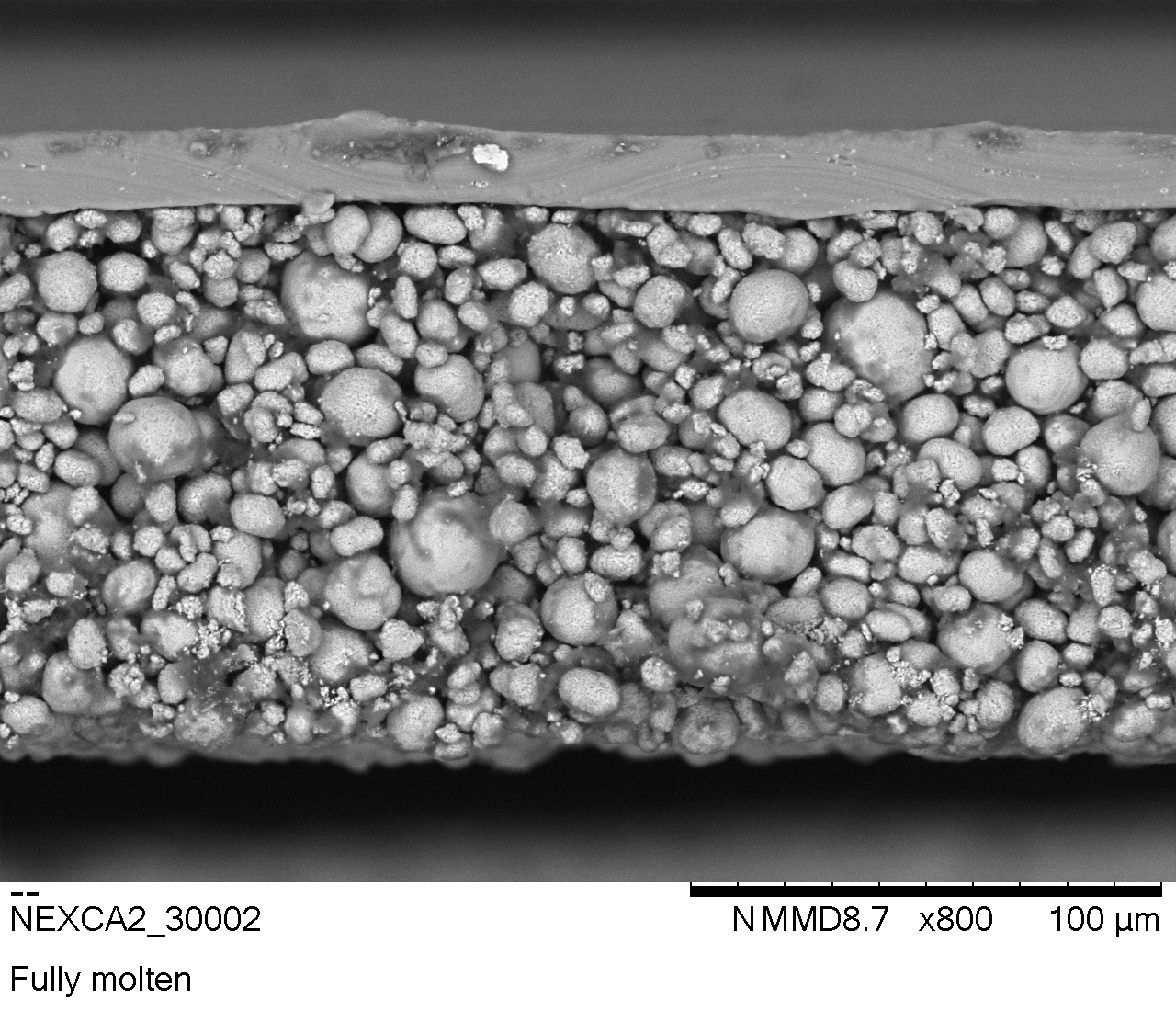

Supplement: Supplementary file 1 [file mmc1.zip › SEM and EDS images/NEXCA2_30002(x800).tif]

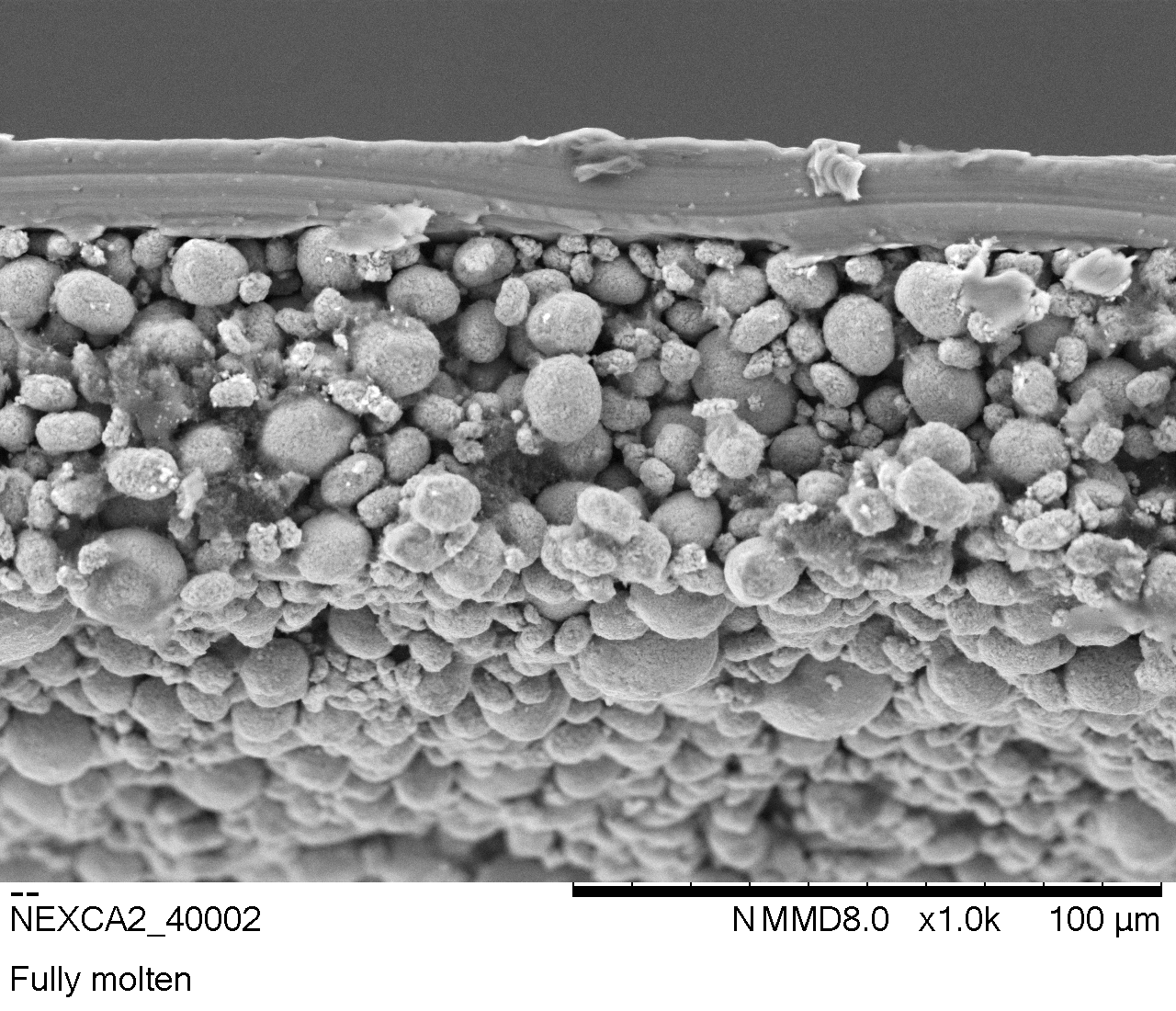

Supplement: Supplementary file 1 [file mmc1.zip › SEM and EDS images/NEXCA2_40002(x1.0k).tif]

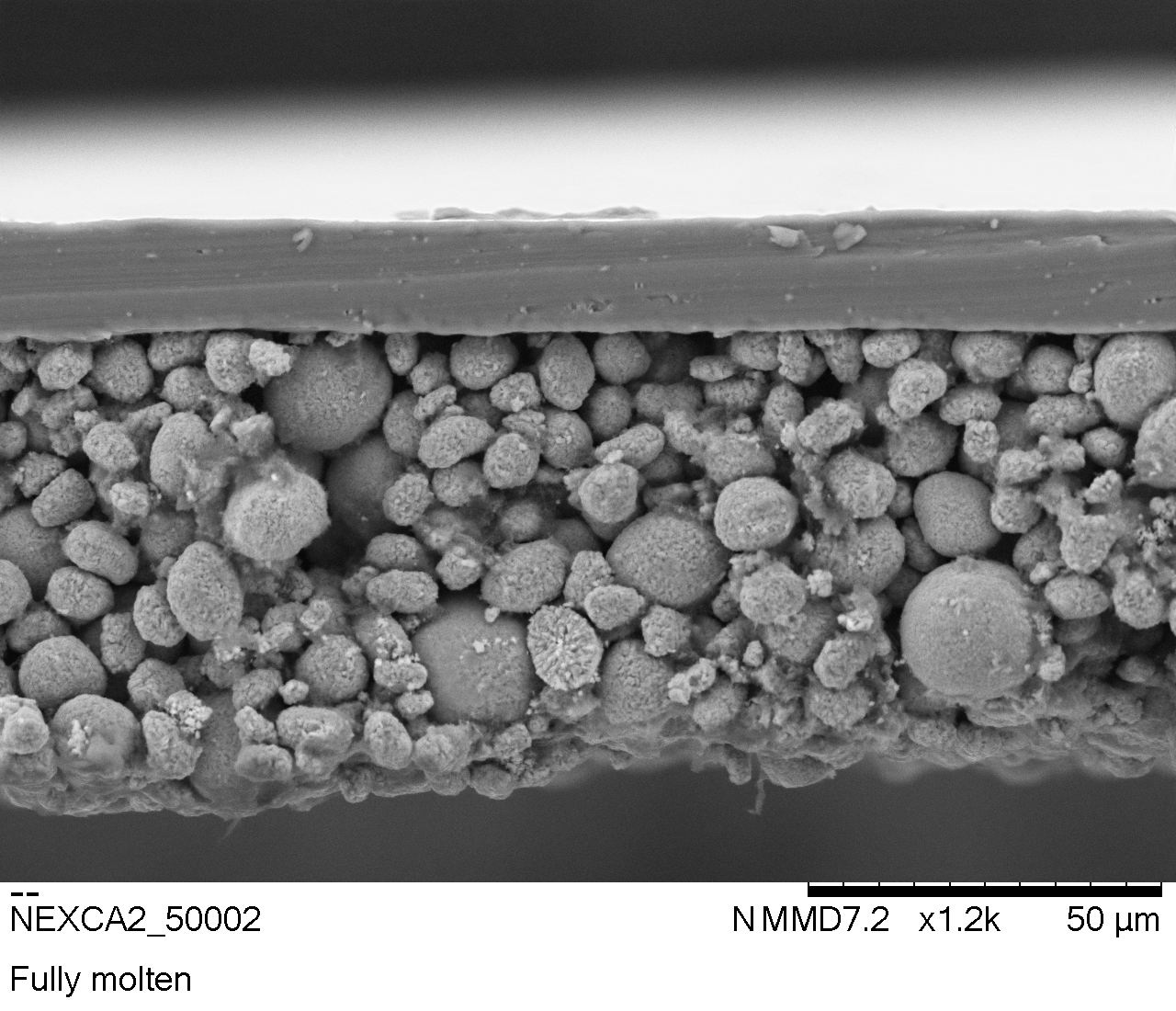

Supplement: Supplementary file 1 [file mmc1.zip › SEM and EDS images/NEXCA2_50002(x1.2k).tif]

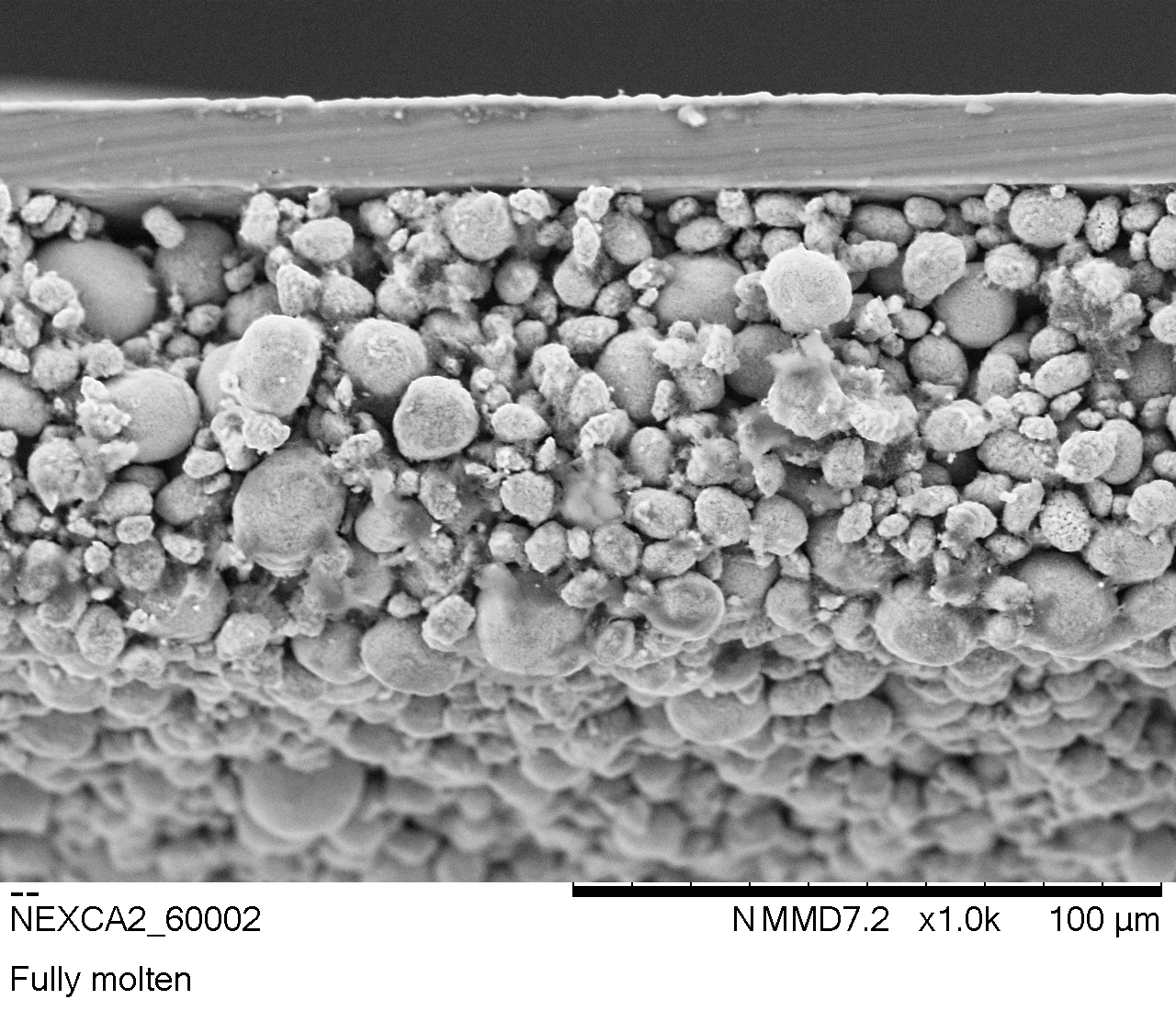

Supplement: Supplementary file 1 [file mmc1.zip › SEM and EDS images/NEXCA2_60002(x1.0k).tif]

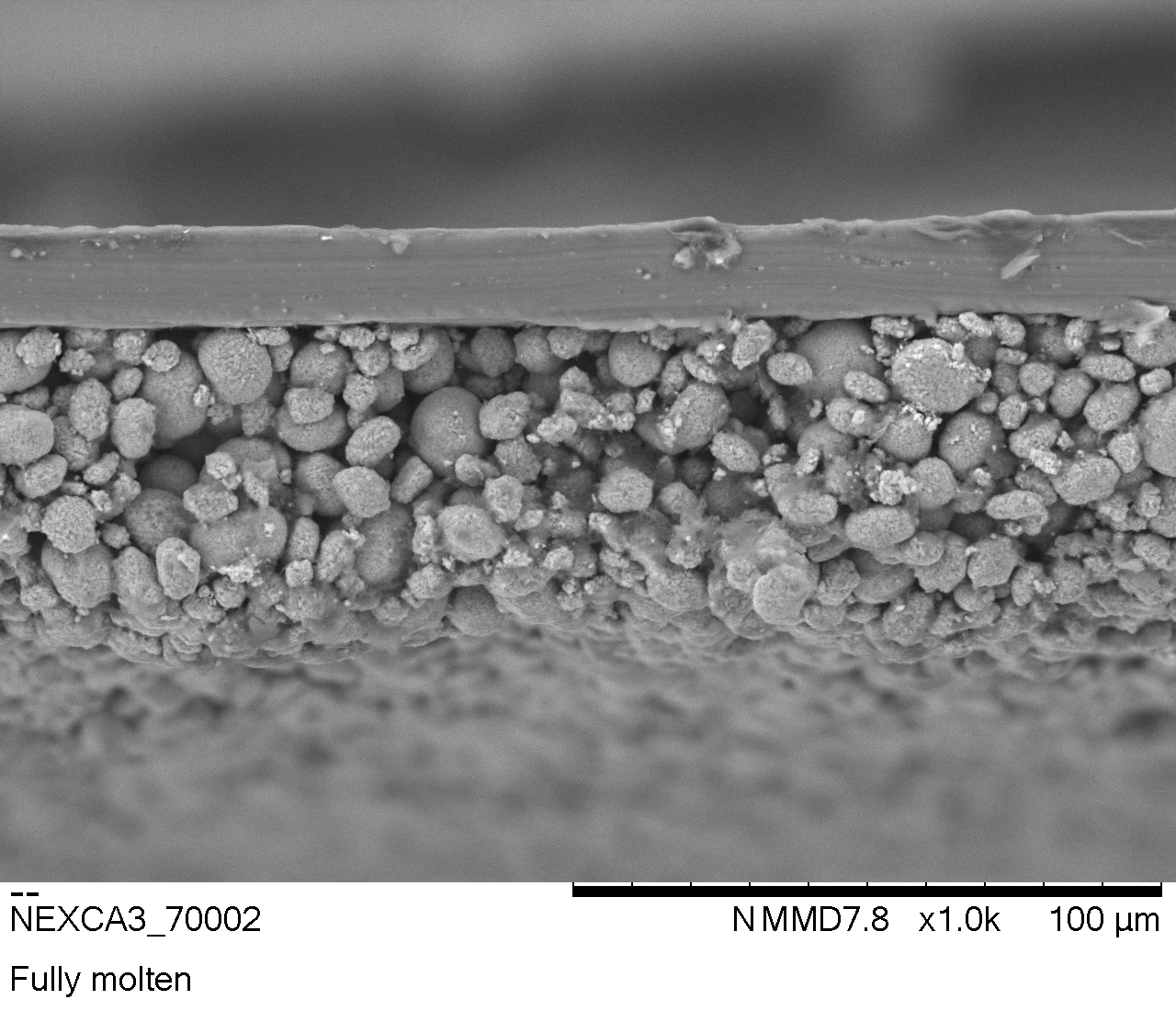

Supplement: Supplementary file 1 [file mmc1.zip › SEM and EDS images/NEXCA3_70002(x1.0k).tif]

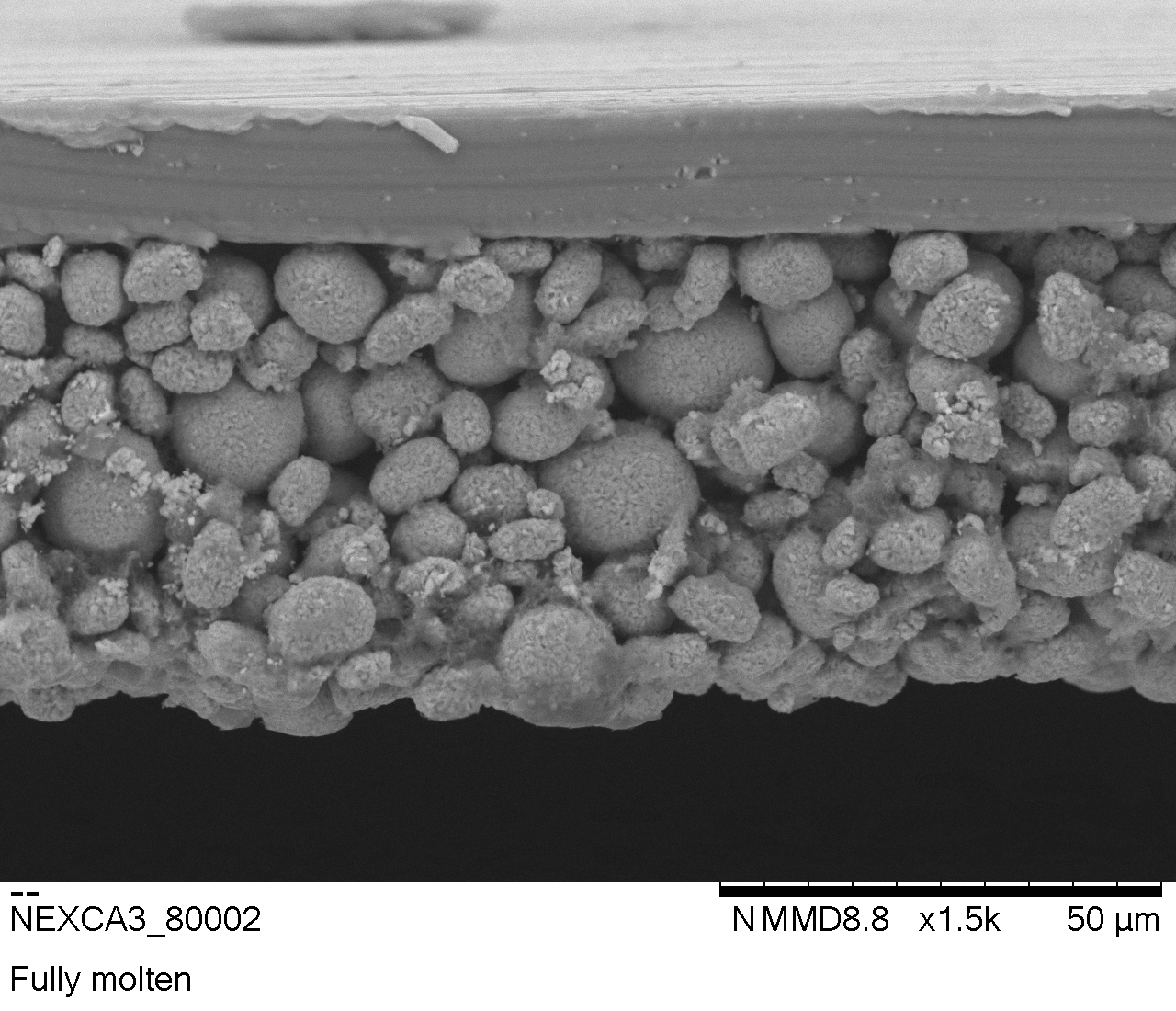

Supplement: Supplementary file 1 [file mmc1.zip › SEM and EDS images/NEXCA3_80002(x1.5k).tif]

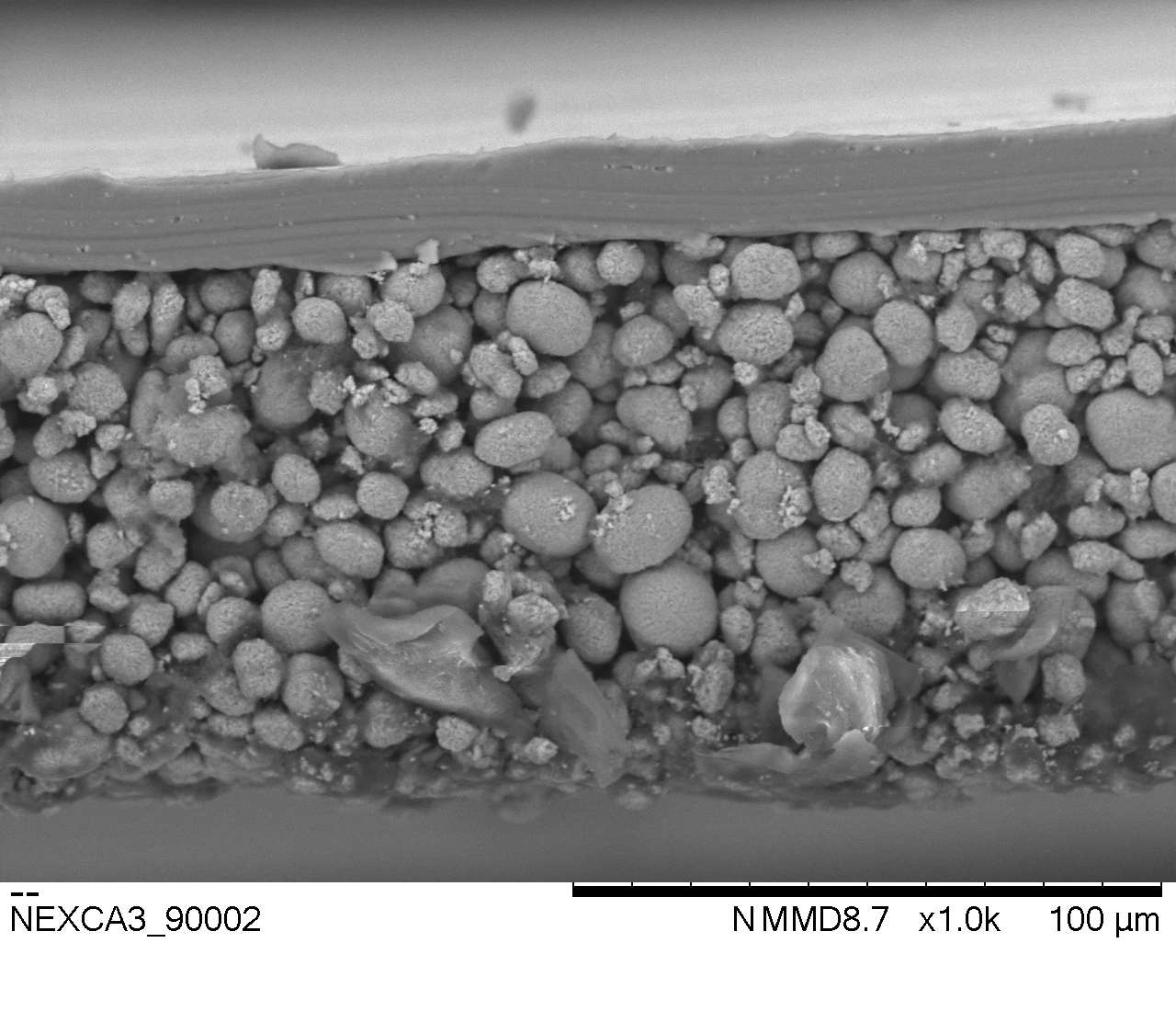

Supplement: Supplementary file 1 [file mmc1.zip › SEM and EDS images/NEXCA3_90002(x1.0k).tif]

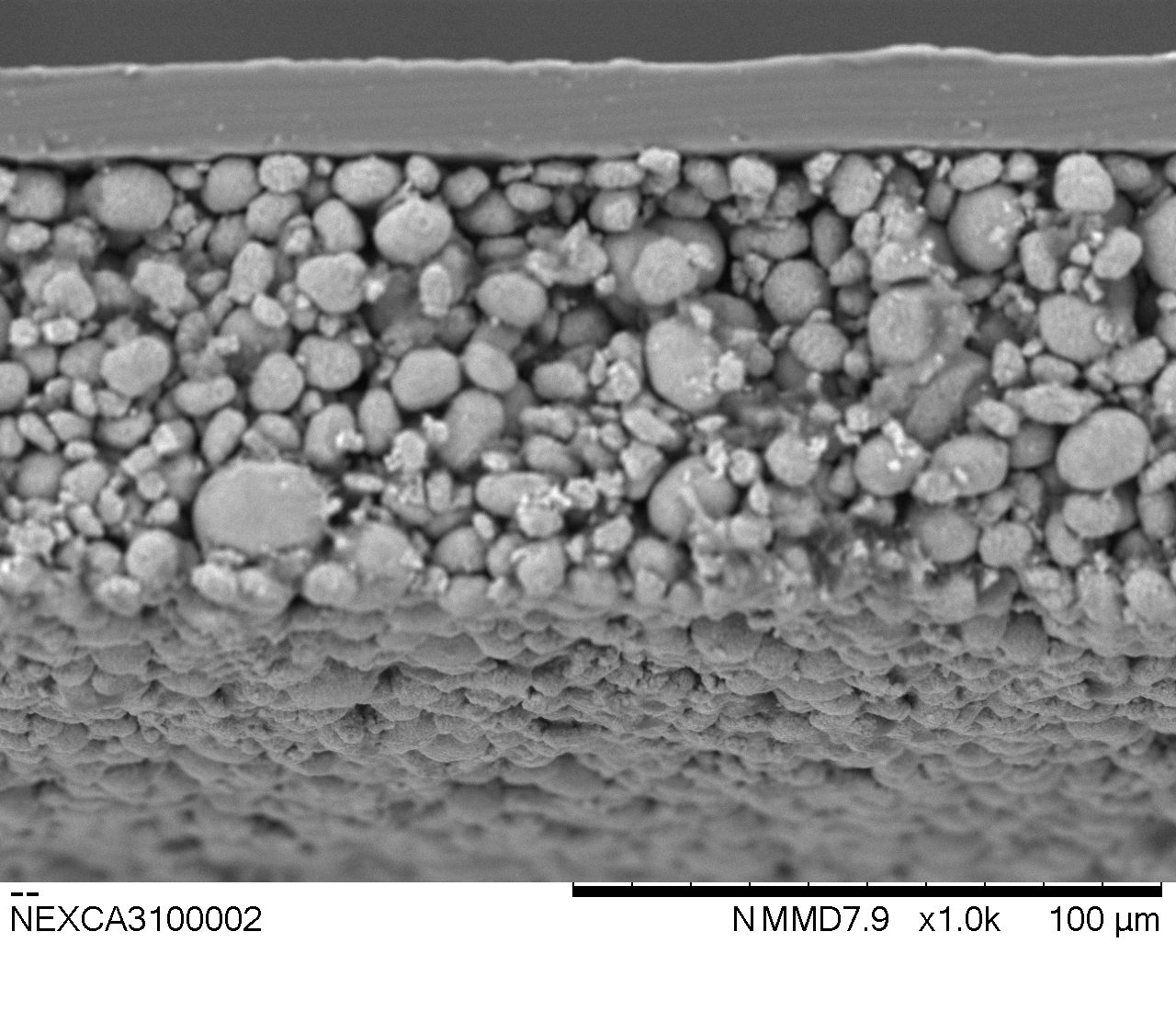

Supplement: Supplementary file 1 [file mmc1.zip › SEM and EDS images/NEXCA3100002(x1.0k).tif]

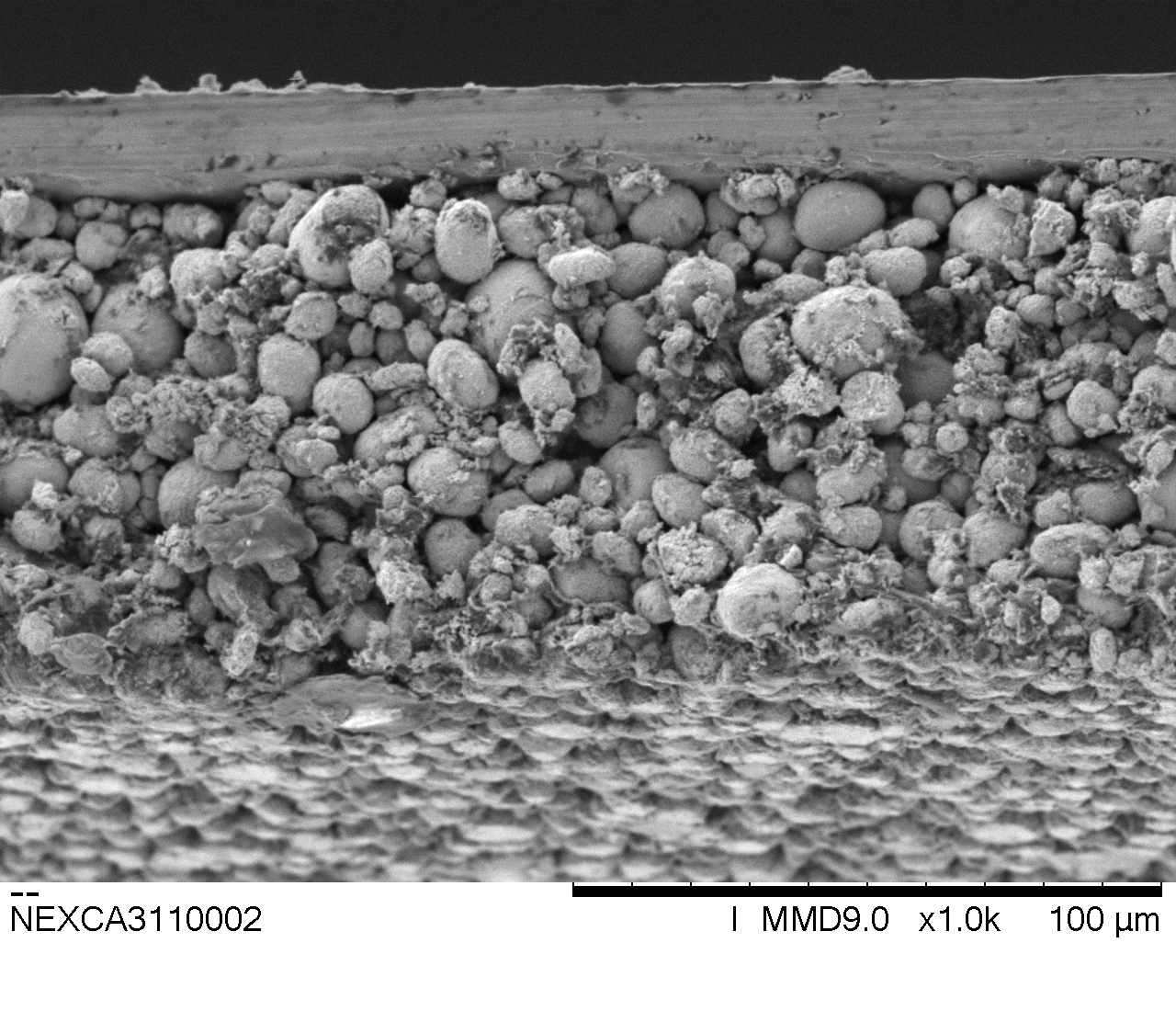

Supplement: Supplementary file 1 [file mmc1.zip › SEM and EDS images/NEXCA3110002(x1.0k).tif]

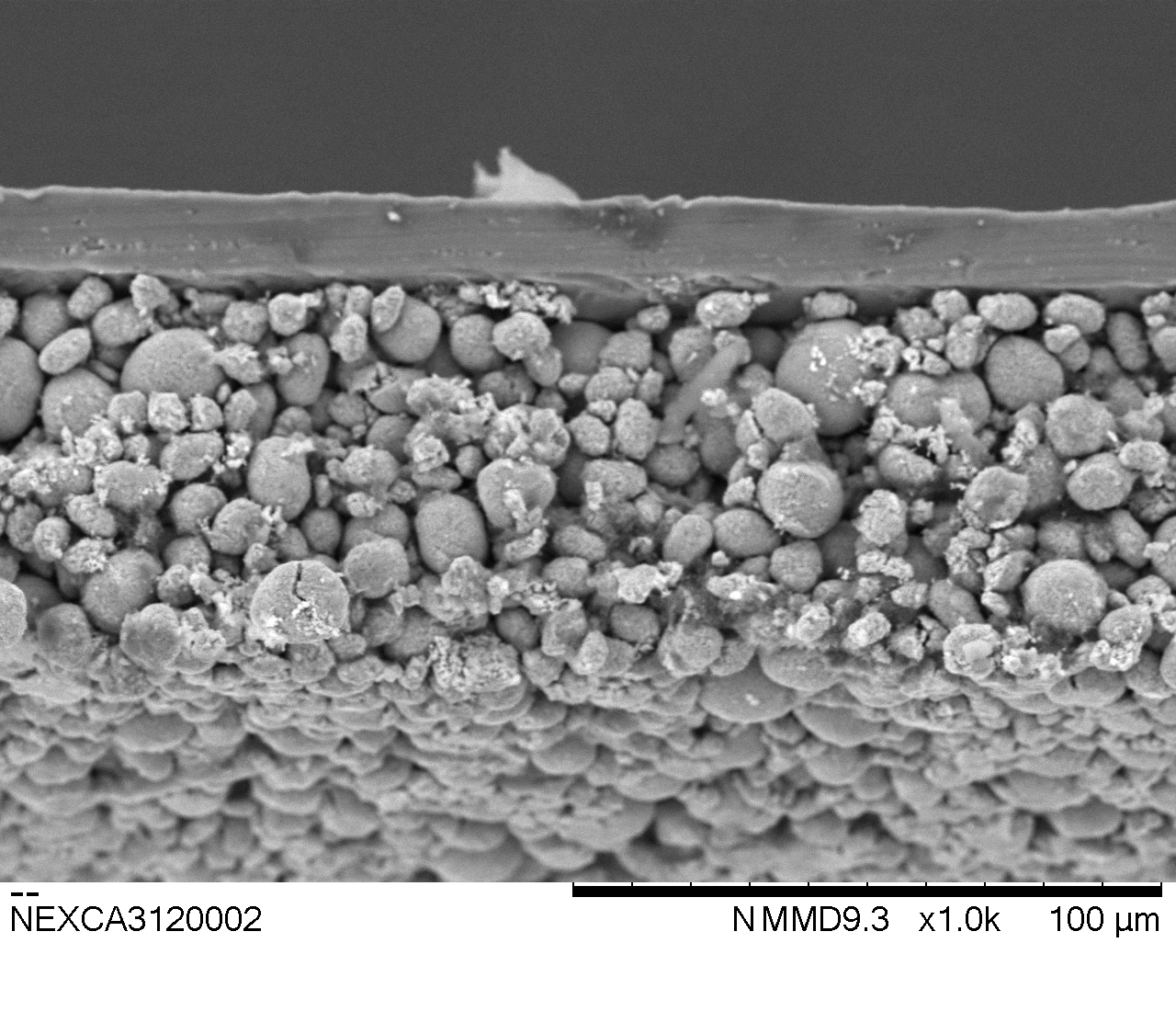

Supplement: Supplementary file 1 [file mmc1.zip › SEM and EDS images/NEXCA3120002(x1.0k).tif]

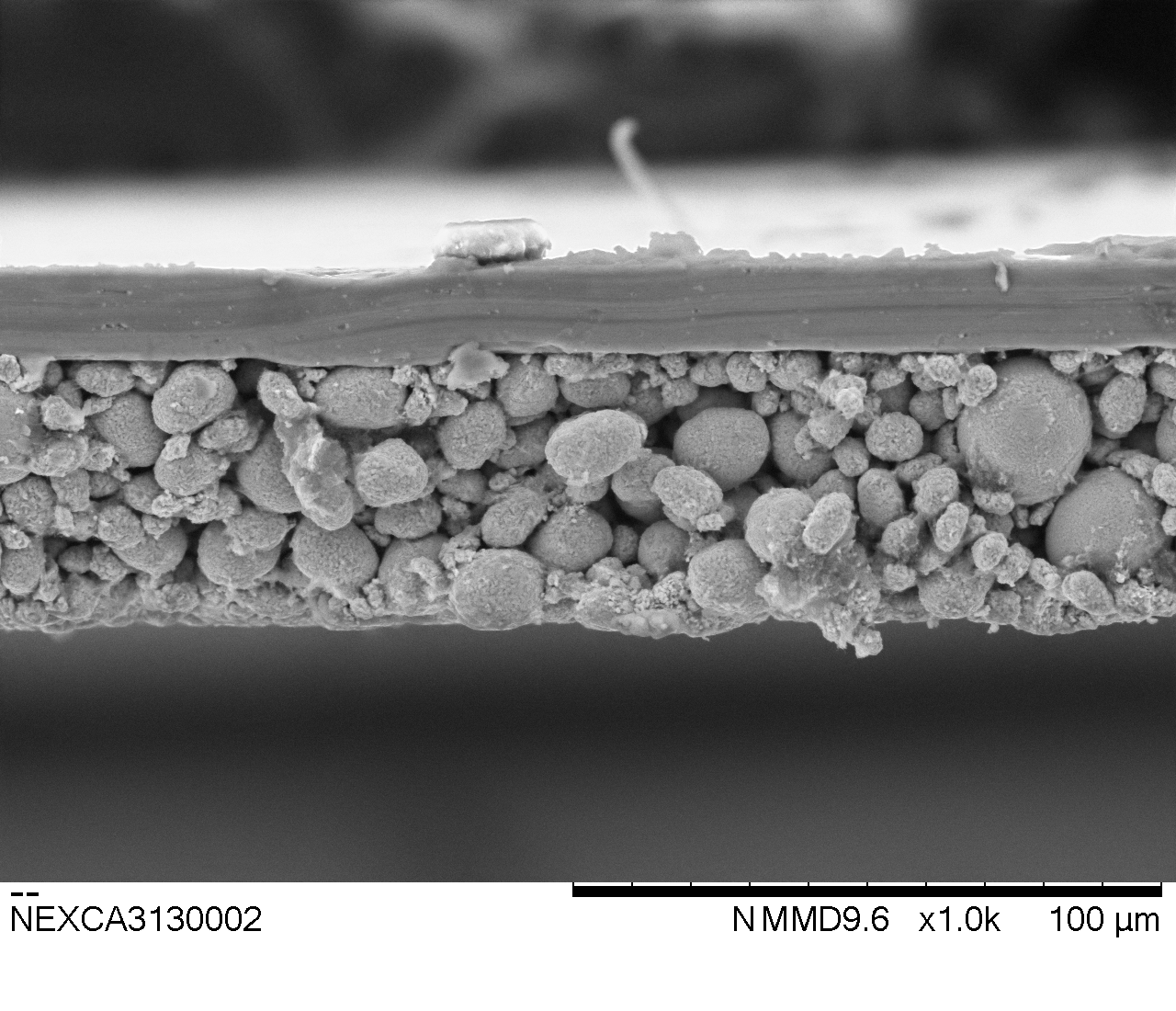

Supplement: Supplementary file 1 [file mmc1.zip › SEM and EDS images/NEXCA3130002(x1.0k).tif]

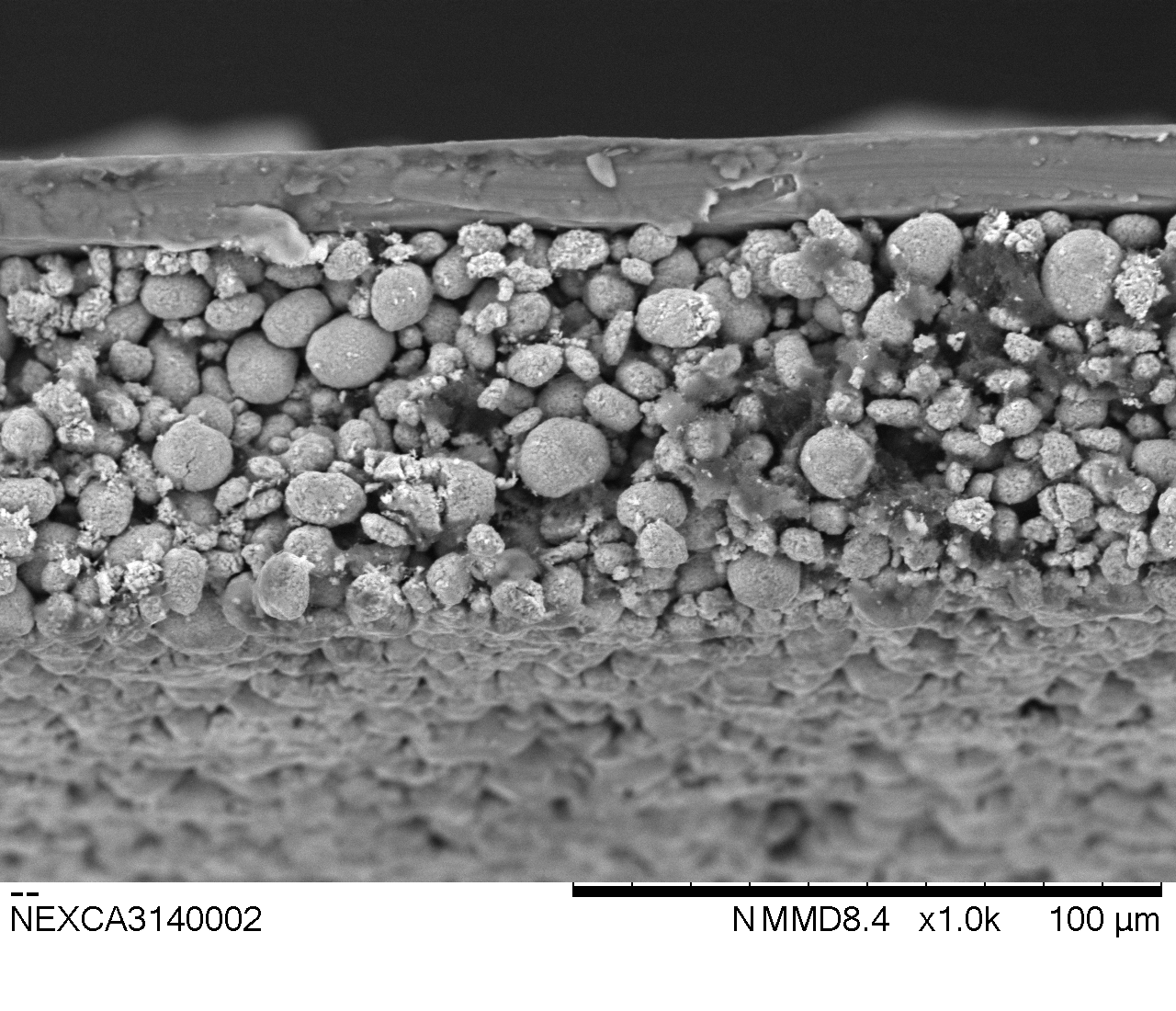

Supplement: Supplementary file 1 [file mmc1.zip › SEM and EDS images/NEXCA3140002(x1.0k).tif]
